# The NATO project: nanoparticle-based countermeasures for microgravity-induced osteoporosis

**DOI:** 10.1038/s41598-019-53481-y

**Published:** 2019-11-20

**Authors:** F. Cristofaro, G. Pani, B. Pascucci, A. Mariani, M. Balsamo, A. Donati, G. Mascetti, G. Rea, A. M. Rizzo, L. Visai

**Affiliations:** 1 Molecular Medicine Department (DMM), Center for Health Technologies (CHT), UdR INSTM, University of Pavia, Viale Taramelli 3/B, 27100 Pavia, Italy; 2 Department of Occupational Medicine, Toxicology and Environmental Risks, Istituti Clinici Scientifici Maugeri S.p.A, IRCCS, Via S. Boezio, 28, 27100 Pavia, Italy; 3 Department of Pharmacological and Biomolecular Sciences, Università degli Studi di Milano, via D. Trentacoste 2, 20134 Milano, Italy; 4 Institute of Crystallography - CNR, via Salaria Km 29.300, 00015 Monterotondo Roma, Italy; 5 Kayser Italia, S.r.l., Via di Popogna, 501, 57128 Livorno, Italy; 6 Italian Space Agency, ASI, Via del Politecnico, 00133 Roma, Italy

**Keywords:** Nanoparticles, Mesenchymal stem cells

## Abstract

Recent advances in nanotechnology applied to medicine and regenerative medicine have an enormous and unexploited potential for future space and terrestrial medical applications. The Nanoparticles and Osteoporosis (NATO) project aimed to develop innovative countermeasures for secondary osteoporosis affecting astronauts after prolonged periods in space microgravity. Calcium- and Strontium-containing hydroxyapatite nanoparticles (nCa-HAP and nSr-HAP, respectively) were previously developed and chemically characterized. This study constitutes the first investigation of the effect of the exogenous addition of nCa-HAP and nSr-HAP on bone remodeling in gravity (1 g), Random Positioning Machine (RPM) and onboard International Space Station (ISS) using human bone marrow mesenchymal stem cells (hBMMSCs). In 1 g conditions, nSr-HAP accelerated and improved the commitment of cells to differentiate towards osteoblasts, as shown by the augmented alkaline phosphatase (ALP) activity and the up-regulation of the expression of bone marker genes, supporting the increased extracellular bone matrix deposition and mineralization. The nSr-HAP treatment exerted a protective effect on the microgravity-induced reduction of ALP activity in RPM samples, and a promoting effect on the deposition of hydroxyapatite crystals in either ISS or 1 g samples. The results indicate the exogenous addition of nSr-HAP could be potentially used to deliver Sr to bone tissue and promote its regeneration, as component of bone substitute synthetic materials and additive for pharmaceutical preparation or food supplementary for systemic distribution.

## Introduction

In healthy adults there are two coordinated processes, predominantly dependent upon the activity of bone-resorbing osteoclasts and bone-forming osteoblasts, which are responsible for continuously remodelling bone tissue. The interrelated processes of bone formation and bone resorption are tightly regulated by both local and systemic signals and by external stimuli. Imbalance in the regulation of these processes may result in osteoporosis, a systemic skeletal condition that is characterized by low bone mass, reduced bone strength and loss of the bone microarchitecture/mineralization. These conditions affect the mechanical properties of bone tissue, leading to an increased risk of fracture due to the decreased density and increased bone fragility^1^.

Conventionally, osteoporosis is defined as primary osteoporosis when associated with sex hormone deficiency and ageing, and as secondary osteoporosis when associated with chronic diseases or disuse. It is known that sedentary lifestyles and immobilization can increase the risk of bone loss, leading to secondary osteoporosis^2^. Additionally, genetic factors, inadequate calcium intake, alcohol and tobacco abuse can increase the risk of osteoporosis^3,4^.

Current clinical therapies include (a) the use of calcium and vitamin D dietary supplements, combined with specific treatments with anti-resorptive or anti-catabolic agents that suppress the activity of osteoclasts (e.g. oestrogen, bisphosphonates, calcitonin, or selective oestrogen receptor modulators), (b) anabolic or bone-forming drugs that stimulate the osteoblast function (e.g. the parathyroid hormone) and (c) anabolic–anti-catabolic agents, such as Strontium ranelate^5^: a dual-mode action drug with interesting properties, due to the presence of strontium ions (Sr^2+^) which accumulate in the mineralized bone matrix, depositing on the apatite surface or substituting calcium ions (Ca^2+^). *In vitro* and *in vivo* studies have established that Sr (administered as Sr chloride, ranelate and lactate) increases bone formation and reduces bone resorption, leading to a gain in bone mass and the improvement of bone mechanical properties in healthy animals and humans^6,7^.

It is also the case, however, that these osteoporotic drugs, mainly Sr ranelate, may present contraindications with long-term use due to bioavailability or toxicity issues although it has been argued that these could be limited by following precise recommendations^8^.

Looking ahead, if we combine medical science, nanotechnology and biochemistry materials, novel nano-bio-systems could be developed for localized and controlled delivery in osteoporosis treatments. In order to successfully support bone regeneration and healing any potential biomaterials need to possess the following features: (a) biocompatible, hydrophilic, non-toxic and biodegradable, so as to allow for the correct regeneration of the tissue; (b) osteoconductive, to encourage cellular adhesion and support the infiltration and proliferation of the new forming tissue; (c) osteoinductive, to recruit progenitor cells and differentiate them into desired lineages; (d) osteogenic and osteointegrated, to guarantee adhesion and bond with the newly forming tissue^9,10^.

In addition to satisfying the majority of these requirements, Hydroxyapatite (HAPs) scaffolds are also very compatible with ionic substitutions and, as such, are an excellent candidate for future clinical applications. Several ions with different properties have been considered as promising therapeutic agents, this list includes Sr ions. Sr-containing hydroxyapatite (Sr-HAP) has been the subject of increasing interest in osteoporotic bone treatment and replacement research, with efficacy in favourable cell attachment, spreading and growth^11^. Several *in vitro* and *in vivo* studies conducted on Sr-HAPs have revealed that they encourage osteoblast proliferation and downregulate osteoclast formation and so increase ALP activity, a renowned biomarker of osteoblast differentiation^12–14^. Furthermore, it has also been shown that Sr-HAP when embedded in scaffold-like-coatings on titanium components, gels, membranes or tablets have similar osteogenic effects of directly administered Sr-HAP in terms of the vitality, proliferation and morphology of cells^15–18^.

The adoption of nano-engineered approaches could help to develop an effective mechanism for providing robust mechanical stability and drug delivery system to accelerate bone regeneration. To this purpose, we have previously developed and characterized stable suspensions of nCa-HAP and nSr-HAP^19,20^ which, combined with the advantageous properties of biomaterial scaffolding at the nanoscale level, could become efficient bone remodeling drugs: at the molecular level, specifically, the added nCa-HAP positively modulated the release of bone-specific markers and enhanced calcified matrix deposition during the osteogenic differentiation of hBM-MSCs^20^. We also evaluated, scanning micro X-ray diffraction and micro X-ray fluorescence, the nucleation, growth, and spatial arrangement of the nCa-HAP nanocrystals. The results revealed the emergence of a complex, heterogeneous and self-organizing process dynamic^20^.

Work on the possibility of controlling bone remodeling through the addition of synthetic nanoparticles could advance the design new drugs in regenerative medicine.

In addition, such studies could have a huge application potential in the prevention/treatment of osteoporosis affecting astronauts or cosmonauts. Disuse osteoporosis is a hot topic in space medicine research, as bone loss among crewmembers is a well-documented condition occurring in the weightless environment of space and due to the reduction of the mechanical stresses on the musculoskeletal system.

It has been determined that cosmonauts, during the course of their posting at the Russian MIR space station, lost the 1–2% of bone mass per month, particularly in the lower halves of their bodies, and have experienced, in some cases, up to a 25% loss of distal tibia mass over a six-month period^21,22^. The decrease in bone density or bone mass has been also been correlated to the presence of increased levels of calcium in the blood because of its release from the hydroxyapatite’s deposit, which is the mineralized inorganic matrix conferring stiffening and strength to the bone tissue^23^. Possible mechanisms of microgravity-induced bone loss include an increased activity of the bone-resorbing osteoclasts and a reduced activity of the bone-forming osteoblasts^24,25^. Once back on earth, however, the negative impact on bone density and strength is only partially reversed: the newly synthesized bone tissue is less mineralized or more porous than the material lost during the disuse period^26^. As a result of these data it is possible for us to hypothesize that longer-term exploration of the Moon and Mars, which would necessitate extended periods in orbit in microgravity conditions, may have more severe and lasting consequences for the bone health of astronauts.

The currently bone-loss countermeasures for astronauts (including intense physical exercise, a balanced diet, and vitamin D supplements while in orbit) cannot by themselves eliminate the risks outlined above. As a result, the development of a pharmacological treatment could be of significant value, as a preventive or remedial therapy.

In order to find countermeasures for microgravity-induced osteoporosis we tested if, and how, nHAP affected the differentiation of mesenchymal stem cells to osteoblasts in 1 g and in microgravity conditions. In the below we present data on cell viability, morphology and differentiation observed before and after treatment with nCa-HAP and nSr-HAP. The results point out for the first time that nHAP could be an active carrier for Sr delivery to bone cells.

## Results and Discussion

“NAnoparticles based countermeasures for Treatment of microgravity induced Osteoporosis” (NATO) is a collaborative project funded by the Italian Space Agency (ASI) to work on the potential of nCa-HAP and nSr-HAP to positively regulate bone remodeling. The primary scientific activities and participating institutions are reported in Table S1. This paper, as indicated above, is the first report of our work on the effect exerted by the exogenous addition of nCa-HAP and nSr-HAP suspensions on hBM-MSCs in 3 different conditions: on the ground at 1 g, in the RPM and in space, onboard the ISS. For the purpose of the experiments, as it has been reported previously^19^, suspensions were produced by dispersing the nCa-HAP and nSr-HAP nanopowders in bovine serum albumin. The replacement of Ca^2+^ with Sr^2+^ in the strontium-substituted hydroxyapatite nanopowder allowed the complete solubility of such alkaline ion in the HAP crystalline lattice, making it suitable as Sr^2+^ ions vector for bone tissue regeneration^19^. The previously biocompatibility studies performed by adding exogenous nanoparticles suspensions to the culture of osteosarcoma cell line SAOS-2 showed the best performance in term of viability and proliferation using the nSr-HAP suspensions with the highest load of Sr^2+^.

### The effect of nanoparticles in suspension on hBM-MSCs cultures in 1 g conditions

#### Cell viability, proliferation, apoptosis and morphological studies

**Figure 1:**
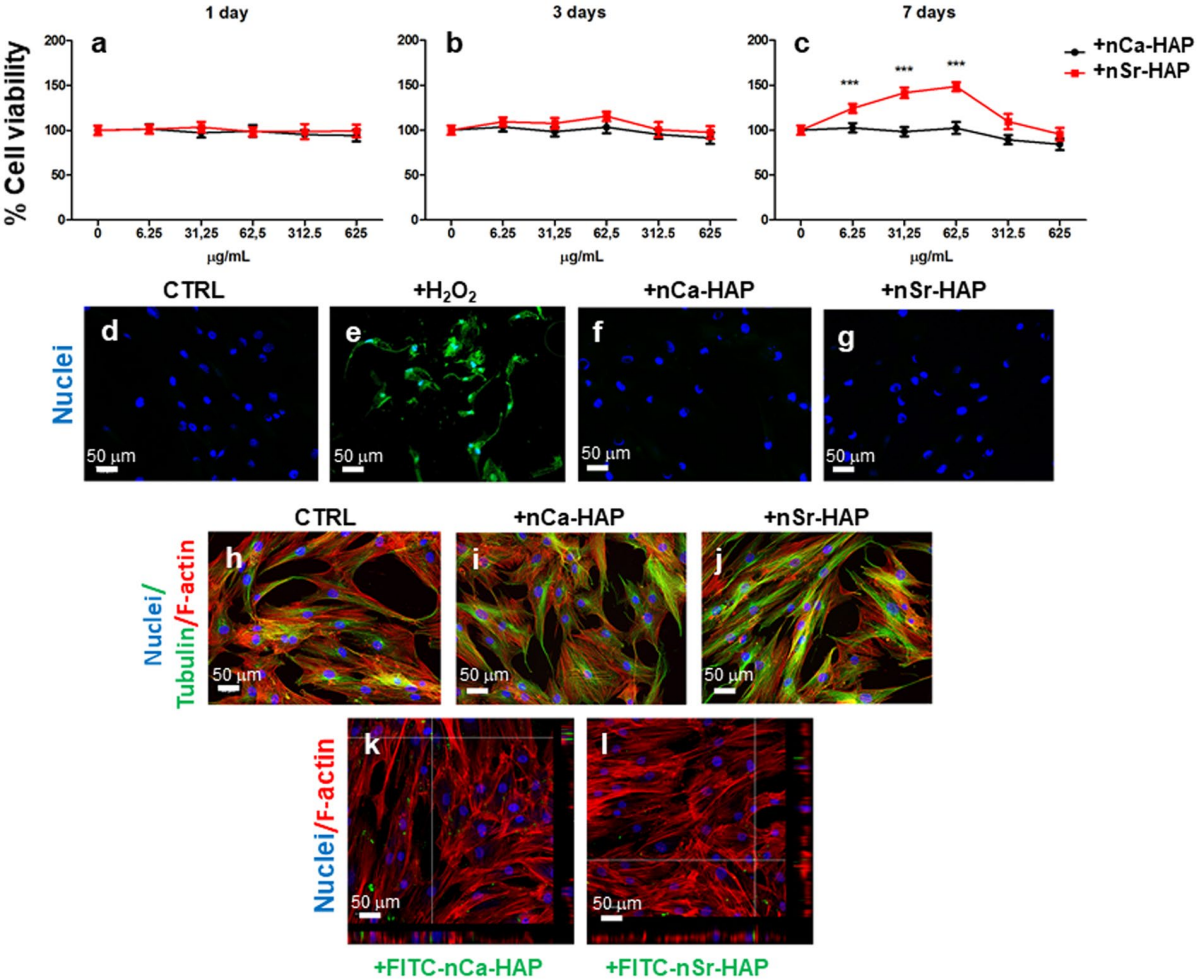
Effect of nanoparticles on the proliferation, apoptosis and morphology of hBM-MSCs in 1 g conditions. Panels (**a–c**) Assessment of dose- and time-dependent responses in terms of cell proliferation of untreated (CTRL) and treated hBM-MSCs with different concentrations of nCa-HAP and nSr-HAP after 1 (**a**), 3 (**b**) and 7 (**c**) days of treatment. The results are expressed as a percentage related to control set at 100%. The presented data are the average ± standard deviations of three measurements in two separate experiments. Statistically significant values are indicated as ***p < 0.001. Panels (**d–g**) Apoptosis evaluation performed at 24 h by PSVue 480 staining on untreated cells (**d**), treated with 100 mM hydrogen peroxide (H_2_O_2_) (**e**), 62.5 µg/mL of nCa-HAP (**f**) or nSr-HAP (**g**) nanoparticles for 24 h. Panels, (**h–j**) Cell morphology was evaluated by staining β-tubulin (green fluorescence) and F-actin (red fluorescence) filaments of either untreated (**h**), nCa-HAP (**i**) or nSr-HAP (**j**) treated hBM-MSCs at 24 h. Panels, (**k–l**) Representative CLSM images and orthogonal projections of hBM-MSCs incubated for 24 h with FITC conjugated nCa-HAP (**k**) or nSr-HAP (**l**). Each scale bar represents 50 μm.

In a previous study^19^, various suspensions of nanoparticles with different concentrations (6.25 μg/ml, 62.5 μg/ml, 625 μg/ml) and time incubations (1, 3 and 7 days) were biologically validated using the osteosarcoma cell line SAOS-2. The present study evaluated the effect of the addition of nanoparticles to the hBM-MSCs cultures on cell viability and proliferation. Furthermore, apoptosis, cell morphology and intracellular uptake of nanoparticles were assessed at 24 hr by CLSM observations. We assessed the dose- and time-dependence effects of exogenous addition of both types of nanoparticles on hBM-MSCs proliferation, by treating cells with 5 different concentrations of nCa-HAP and nSr-HAP for 1, 3 and 7 days in comparison with untreated cells (Fig. 1). The results showed all tested nCa-HAP concentrations did not significantly decrease cell viability in samples treated for 1 and 3 days compared to untreated samples (Fig. 1, panels a and b). The substitution of Ca^2+^ with Sr^2+^ did not compromise cell viability, rather longer incubation times (up to 7 days) promoted an increase of cell proliferation (20–50%) in a concentration-dependent manner up to 62.5 μg/mL (Fig. 1, panel c). In this context, the 62.5 μg/mL was the safest and most efficient concentration of nSr-HAP to be used for hBM-MSCs cultures, as it has been previously observed in the SAOS-2 cell line^19^.

Untreated cells (Fig. 1, panel d) and H_2_O_2_ treated cells (positive CTRL, Fig. 1, panel e), incubated with a 62.5 μg/mL nCa-HAP (Fig. 1, panel f) or nSr-HAP (Fig. 1, panel g) were stained with PSVue 480 to assess apoptosis after a 24 h incubation, as indicated in the materials and methods section. No differences were observed with other nanoparticle concentrations (data not shown). As expected, apoptosis was observed in the sample treated with H_2_O_2_, showing an intense green fluorescence, whereas no staining was detected in the treated and untreated samples with nanoparticles.

Untreated and treated samples with 62.5 μg/mL nCa-HAP or nSr-HAP suspensions were observed by CLSM after proper staining, in order to evaluate cell morphology at 24 h. No significant differences in the cytoskeleton were observed between the untreated cells (Fig. 1, panel h) and the cells treated with nCa-HAP (Fig. 1, panel i) or nSr-HAP (Fig. 1, panel j) exhibiting the typical fibroblast-like morphology of hBM-MSCs. In addition, the FITC conjugated nanoparticles (FITC-nPS) uptake was evaluated at 24 h (Fig. 1, panels k,l) by CLSM analysis. Interestingly, both types of FITC-NPs were detected inside the cells with no significant differences between the nCa-HAP (Fig. 1, panel k) or nSr-HAP (Fig. 1, panel l) treatments. Further studies are needed to evaluate the uptake mechanism and its effect on cell differentiation.

The observations made by using an Olympus camera connected to an optical microscope of the hBM-MSCs at 24 h (Fig. S1, panels b and c) and 72 h (Fig. S1, panels e and f) did not reveal any differences in growth, demonstrating that the exogenous addition of nanoparticles, when compared to untreated cells (Fig. S1, panels a and d), did not hamper the proliferation or physiological adhesion processes. The results obtained at 72 h were also confirmed by TEM images (Fig. S1, panels g–i).

#### Differentiation studies

Since the *in vitro* stem cells differentiation into osteoblasts is completed in approximately 28 days in the presence of an osteogenic medium (OM), we also evaluated the effect of the addition of both types of nanoparticles to hBM-MSCs cultured for 28 days in proliferative medium (PM, without osteogenic factors) in 1 g conditions. The rationale for this experiment was to clarify the role of the nanoparticles as potential osteogenic factors for stem cell differentiation to osteoblasts. To this end, the specificity of Sr^2+^ on the spatiotemporal evolution of differentiation towards osteoblasts was tested by evaluating ALP activity at 2 time points (8 and 28 days). Moreover, the quantitative bone gene marker expression (qRT-PCR), and the calcified bone matrix deposited (alizarine red staining, ELISA assay and CLSM), either in PM or OM conditions, were detected after 28 days of treatment.

In OM culture conditions, a significant increase in ALP activity in the samples treated with either 31.25 or 62.5 μg/mL nSr-HAP (p < 0.01) were observed in comparison with untreated samples, while no differences were found in cells treated with nCa-HAP (p > 0.05) (Fig. S2). At higher nSr-HAP concentrations, a significant decrease of ALP activity and a higher number of adipocytes were revealed. In particular, adipocytes were recognized after 28 days of treatment of hBM-MSCs with nSr-HAP at concentrations of 312.5 and 625 μg/mL: in fact, these treated cells presented large lipid droplets in the cytoplasm confirmed by specific staining with an Oil red O dye (manuscript in preparation). ALP activity was not modified by the addition of nCa-HAP and nSr-HAP (p > 0.05) in PM conditions (Fig. S2).

**Figure 2:**
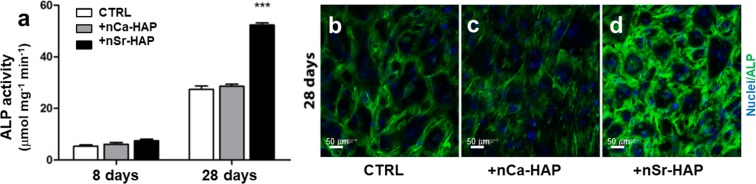
Effect of nanoparticles on ALP activity and protein immunolocalization in 1 g conditions. Panel (**a**) ALP specific activity of untreated, nCa-HAP or nSr-HAP treated hBM-MSCs for 8 and 28 days in an osteogenic medium (OM). Data are presented as the average ± standard deviation of three measurements in two separate experiments. Statistically significant values are indicated as ***p < 0.001. Panels (**b–d**) Representative CLSM images of ALP immunostaining of untreated (**b**), nCa-HAP (**c**) or nSr-HAP (**d**) treated hBM-MSCs cultured for 28 days in OM. Magnification 40X, scale bars represent 50 μm.

Since the treatment of hBM-MSCs with 62.5 μg/mL nanoparticles determined the best performance in terms of cell proliferation and ALP activity during differentiation, this concentration was used in our subsequent studies in OM culture conditions. Figure 2, panel a reports the ALP values of the untreated and nCa-Hap or nSr-HAP treated hBM-MSCs differentiated for 8 and 28 days in OM culture conditions. Data at 28 days confirmed the previous observations, whereas no significant differences were observed at day 8, indicating that the early differentiation process is probably unaffected by the addition of exogenous nanoparticles. ALP immunolocalization displayed a more intense, diffuse green fluorescence in nSr-HAP treated hBM-MSCs (Fig. 2, panel d) when compared to the untreated and nCA-HAP treated cells (Fig. 2, panels b and c).

Cell viability during the differentiation phase was monitored at 8 and 28 days using a 62.5 μg/mL concentration for both types of nanoparticles. We observed increased proliferation in cells treated with nSr-HAP in OM culture conditions when compared to the untreated or nCa-HAP treated samples (data not shown).

**Figure 3:**
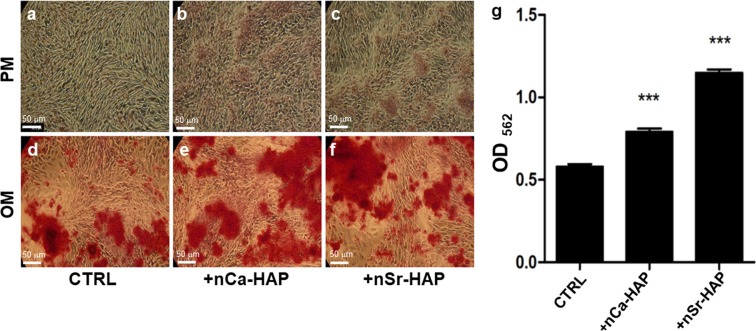
Effect of nanoparticles on calcium deposits evaluated by Alizarin Red staining in 1 g conditions. Panels (**a–f**) Representative images performed with an optical microscope of untreated (panels** a** and **d**), nCa-HAP (panels **b** and **e**) or nSr-HAP (panels **c** and **f**) treated hBM-MSCs, cultured for 28 days in PM (panels **a–c**) and OM (panels **d–f**) conditions and stained with Alizarin Red S for calcium deposits visualization. Magnification 10X, scale bars represent 50 μm. Panel (**g**) Quantitative evaluation of Alizarin Red staining after cetylpyridinium dissolution of untreated (CTRL), nCa-HAP or nSr-HAP treated hBM-MSCs cultured for 28 days in OM culture conditions (by optical density reading at a λ562). The data are presented as an average ± standard deviation of three measurements in two separate experiments. Statistically significant values are indicated as ***p < 0.001.

We further evaluated the effect of nCa-HAP or nSr-HAP on the levels of calcium deposited after 28 days of treatment in PM or OM culture conditions by Alizarin Red staining and its quantification after cetylpyridinium dissolution (by optical density reading at λ562), as shown in Fig. 3. In contrast to PM conditions (Fig. 3, panels a–c), the Alizarin red staining was more intense in the samples cultured in OM culture conditions (Fig. 3, panels d–f). Moreover, in the nCa-HAP and nSr-HAP treated hBM-MSCs, we observed a statistically higher number of calcium deposits in comparison with the untreated cells; this data was supported by an absorbance reading performed at 562 nm after cetylpyridinium dissolution (Fig. 3, panel g) (p < 0.001). These data suggest that nanoparticles, and in particular nSr-HAP, have a positive effect on bone matrix mineralization.

**Figure 4:**
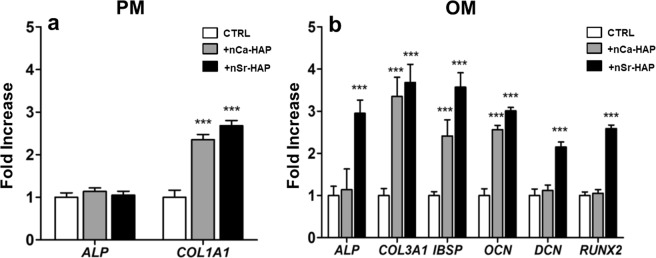
Effect of nanoparticles on gene expression of bone specific markers as determined by qRT-PCR in 1 g conditions. Untreated, nCa-HAP and nSr-HAP treated hBM-MSCs were cultured for 28 days in PM (panel **a**) and OM (panel **b**) conditions, respectively. The graph indicates the fold increase of gene expression related to untreated cells. Data are presented as average ± standard deviation of three measurements in two separate experiments. Statistically significant values are indicated as ***p < 0.001.

To get deeper insights into the ability of nCa-HAP or nSr-HAP to induce differentiation of hBM-MSCs into osteoblasts, we also tested the regulatory mechanisms underlying this process by analyzing the expression profile of marker genes in PM and OM conditions for 28 days. Increased expression levels of the *COL1A1* gene, encoding the main protein occurring in connective tissues and bone matrix, was detected in PM conditions in the presence of both type of nanoparticles (p < 0.001). Furthermore, a positive correlation between gene expression and enzyme activity was revealed concerning the *ALP* gene (Fig. 4, panel a). Moreover, in the same conditions, the expression of the marker gene *COL3A1*, *IBSP*, *OCN DCN*, and *RUNX2* did not show any significant difference in untreated and nCa-HAP or nSr-HAP treated samples (data not shown). On the other hand, in OM culture conditions, we detected an increase in gene expression of *COL3A1*, *IBSP*, and *OCN* in both treated samples although *ALP*, *DCN* and *RUNX2* showed a significant fold increase only for samples treated with nSr-HAP (Fig. 4, panel b) (p < 0.001). All these genes codify proteins involved in the osteogenic differentiation process. Runx2 is a transcription factor required for osteoblast differentiation. It is interesting to note that Runx2-null mice are completely deprived of osteoblasts^27^. After Runx2 expression, osteoblast progenitors acquire bone-specific ALP properties, responsible for a high concentration of phosphate at the mineral deposition site. The late stage of osteoblast differentiation is characterized by high expression of proteins involved in bone matrix deposition, such as type-I and -III collagen (COL1A1 and COL3A1, respectively), decorin (DCN), osteocalcin (OCN), which is almost exclusively expressed in bone, and by the production of non-collagenous proteins such as bone sialo protein (IBSP)^20,28,29^.

**Figure 5:**
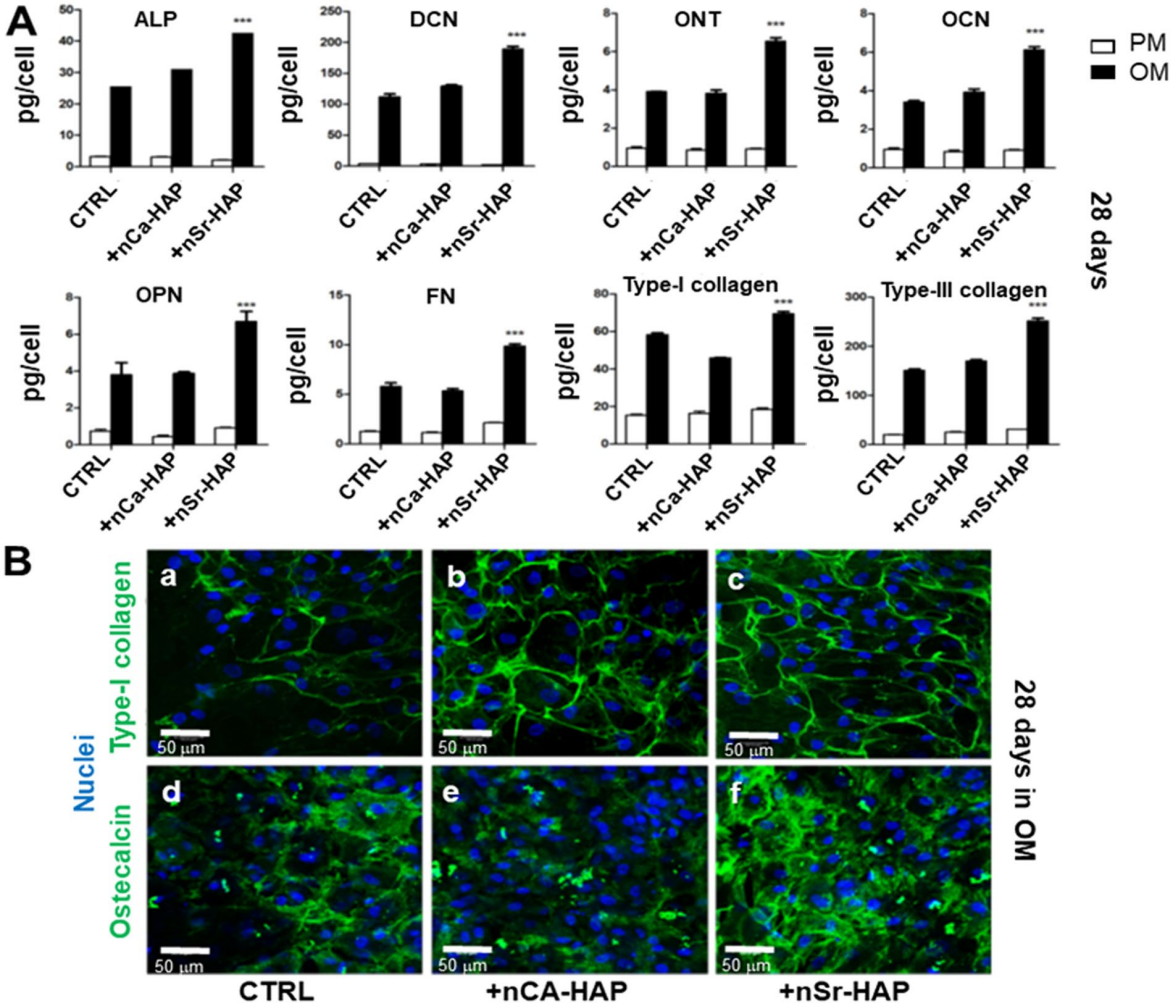
Effect of nanoparticles on bone matrix proteins deposition in 1 g conditions. (**A**) Extracellular matrix proteins deposits (ALP, DCN, ONT, OCN, OPN, FN, Type-I and -III collagens) of untreated, nCa-HAP or nSr-HAP treated hBM-MSCs, cultured in PM and OM culture conditions for 28 days as determined by ELISA assay. Data are presented as average ± standard deviation for three measurements in two separate experiments. Statistical significance values are indicated as *p < 0.05, **p < 0.01, and ***p < 0.001; (**B**) Representative CLSM images for type-I collagen and osteocalcin deposited by untreated (panels **a** and **d**), nCa-HAP (panels **b** and **e**) or nSr-HAP (panels **c** and **f**) treated hBM-MSCs cultured for 28 days in OM. Magnification 20X, scale bars represent 50 μm.

Finally, deposits of specific bone matrix proteins were evaluated after 28 days in untreated and nCa-HAP or nSr-HAP treated hBM-MSCs cultured in PM and OM culture conditions by ELISA immunoassay (Fig. 5). Results indicated the expression levels of all tested proteins increased in OM culture conditions in nSr-HAP treated samples compared to the untreated samples (Fig. 5A). On the contrary, no differences were observed in PM culture conditions (Fig. 5A). Furthermore, Type-I collagen and osteocalcin were also analyzed by immunofluorescence experiments in OM culture conditions (Fig. 5B), revealing a more intense and diffuse green fluorescence for both proteins in nSr-HAP treated samples (Fig. 5B, panels c and f) in comparison with untreated (Fig. 5B, panels a and d) and nCa-HAP treated cells (Fig. 5B, panels b and e). This conclusion is supported by the quantitative data collected by ELISA.

The analyzed ECM proteins are organic components of bone matrix and impact on bone remodeling and formation. Type-I and type-III collagen are the major components of bone ECM and, through their organization in fibrils and fibers, they constitute the supporting structure of the bone matrix. OPN and FN are known to play important role in cell attachment and proliferation^30^, whereas ONT, OCN and DCN interact with mineral deposits inside the matrix^31^. The increased deposition of these proteins by the hBM-MSCs treated with nSr-HAP indicates the positive impact that these nanostructures have on the organic portion of the bone matrix as well as on the mineral part, as shown previously.

The results of our cell differentiation studies in 1 g conditions allowed us to set up the Simulated Microgravity and Space Flight experiments by adding nCa-HAP or nSr-HAP at 62.5 μg/mL in the osteogenic culture medium (OM). Furthermore, since the maximum time provided for the NATO payload experiment on the ISS to perform the cell differentiation studies in the absence and presence of each type of nanoparticles was 88 h, an identical period of incubation was used for the Simulated Microgravity (with RPM) experiments.

### nCa- and nSr-HAP as potential countermeasures for bone loss induced by microgravity

#### Pre-flight experiment in simulated microgravity with RPM

**Figure 6:**
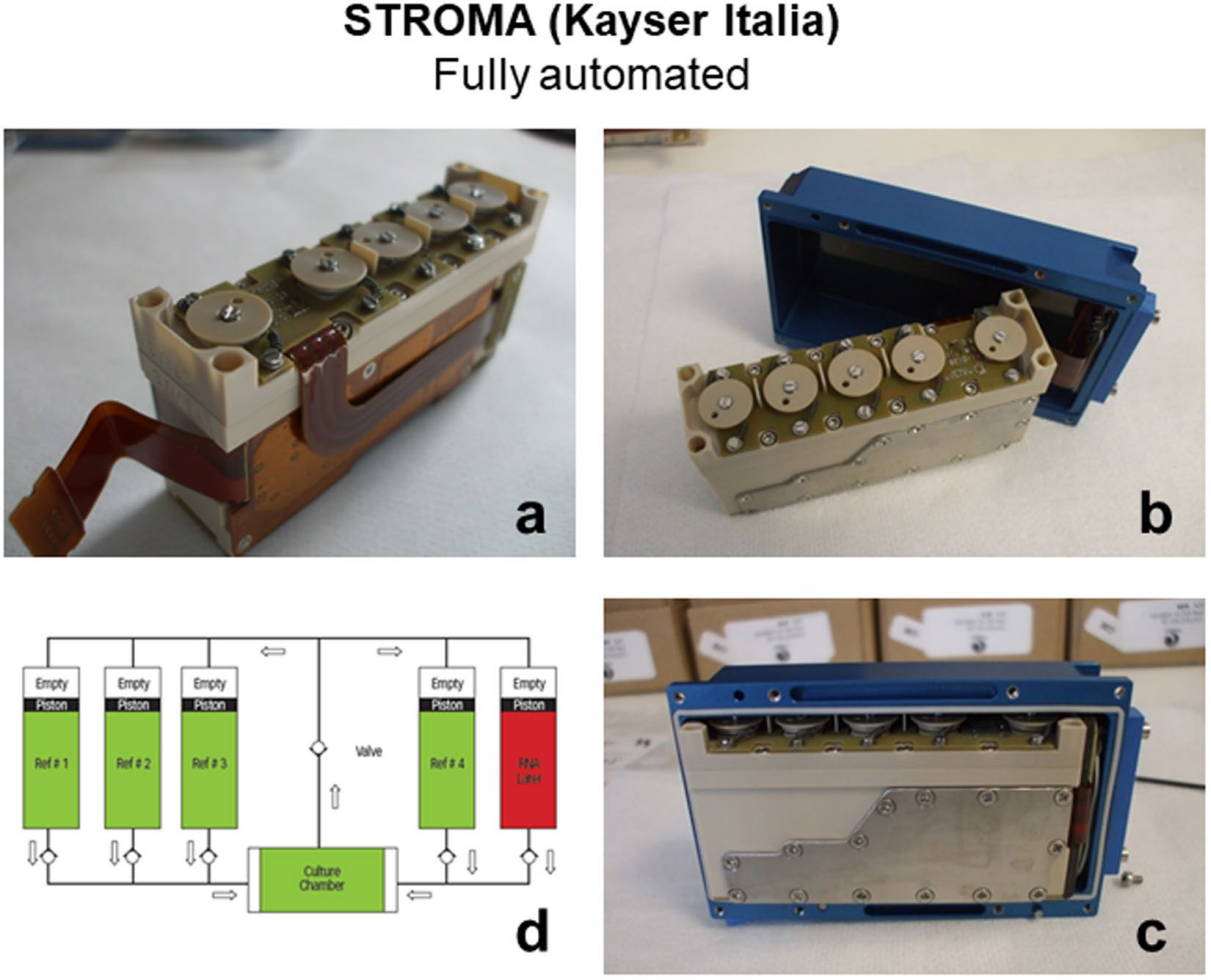
STROMA EU used for NATO space flight. Panels **a–d**: STROMA EU (panel **a**), STROMA EU cased within the KIC (in blue, panels **b** and **c**) and the fluidic system scheme (panel **d**).

STROMA Hardware (HW) Experimental Units (EU) were used to perform the NATO experiments onboard the ISS (Fig. 6, panel a)^32^. This type of hardware was developed for the STROMA experiment that investigated mice BMSCs and was launched in 2003 on the STS-107 Shuttle. The same type of HW was also used in 2006 (Soyuz 12 S), 2007 (Foton) and 2010 (Progress 40 P). In general, the EU are equipped with reservoirs to be filled with chemicals (culture medium, washing buffer, fixatives) and a culture chamber allowing for the growth of adherent cells on Thermanox. This system is capable of performing automatic 2D cell culturing in microgravity thanks to a microfluidic system (Fig. 6, panel d). Each STROMA EU used by the NATO experiment was accommodated into the KIC (in blue, Fig. 6, panels b and c) which contains programmable electronics, allowing for a predefined timeline of the experimental protocol. At the end of the experiment, the EU can be stowed in flight at controlled temperatures, down to −80 °C. After stowage and re-entry to Earth, the 2D cell cultures of the STROMA EU can be analyzed by microscopy techniques and by molecular biology-based approaches for genomic, transcriptomic and proteomic studies.

**Figure 7:**
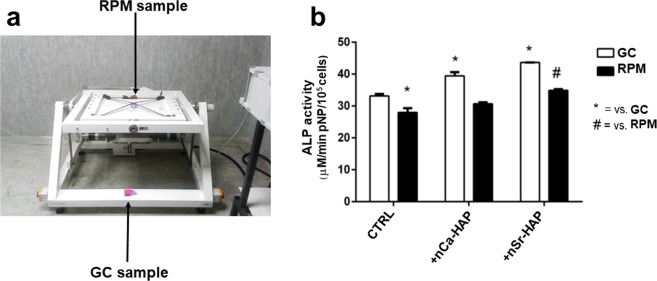
Effect of nanoparticles on ALP activity of hBM-MSCs cultured in STROMA EU in RPM. Panel (**a**) The RPM was used on ground to simulate microgravity in order to set up the biological conditions for the ISS NATO space flight experiment. Panel (**b**) ALP enzymatic activity of hBM-MSCs cultured in OM culture conditions without (CTRL) or with the addition of nCa-HAP and nSr-HAP nanoparticles for 88 h in RPM and in 1 g ground control (GC); time and concentration were the same of the real space flight. Data are presented as average ± standard deviations of three measurements. Statistical significance values are indicated as *p < 0.05 *vs* ground untreated cells (CTRL, white bar), and ^#^p < 0.05 RPM untreated cells (CTRL, black bar), in absence of nanoparticles.

Preparations of the NATO Space Flight Experiments included an Experimental Sequence Test (EST) to assess the assembly development of the STROMA EUs, the loading of the reservoirs and the hBM-MSCs culture in simulated microgravity conditions using the Random Positioning Machine (RPM)^33^ (Fig. 7, panel a).

Preliminary experiments were then carried out to determine the variables induced by both the microgravity conditions and by the STROMA EU culture system (Fig. S3). Once the exposure to simulated microgravity was over, Thermanox coverslips were removed from the culture chambers and the expression of bone gene markers was analyzed (Fig. S3). After 88 h of exposure to simulated microgravity, and as expected, the expression of osteoblastic gene markers was significantly reduced in the RPM samples, as compared to the GC samples (Fig. S3). Further experiments were performed on RPM samples in OM culture conditions with/without nCa-HAP and nSr-HAP to evaluate cell viability (data not shown) and ALP activity (Fig. 7, panel b). Cell viability was not reduced by the addition of both types of nanoparticles and the nSr-HAP treatment was able to counteract the effects of microgravity on ALP activity by greatly promoting to the same level of GC untreated samples (Fig. 7, panel b).

#### International space station (ISS) experiment

**Figure 8:**
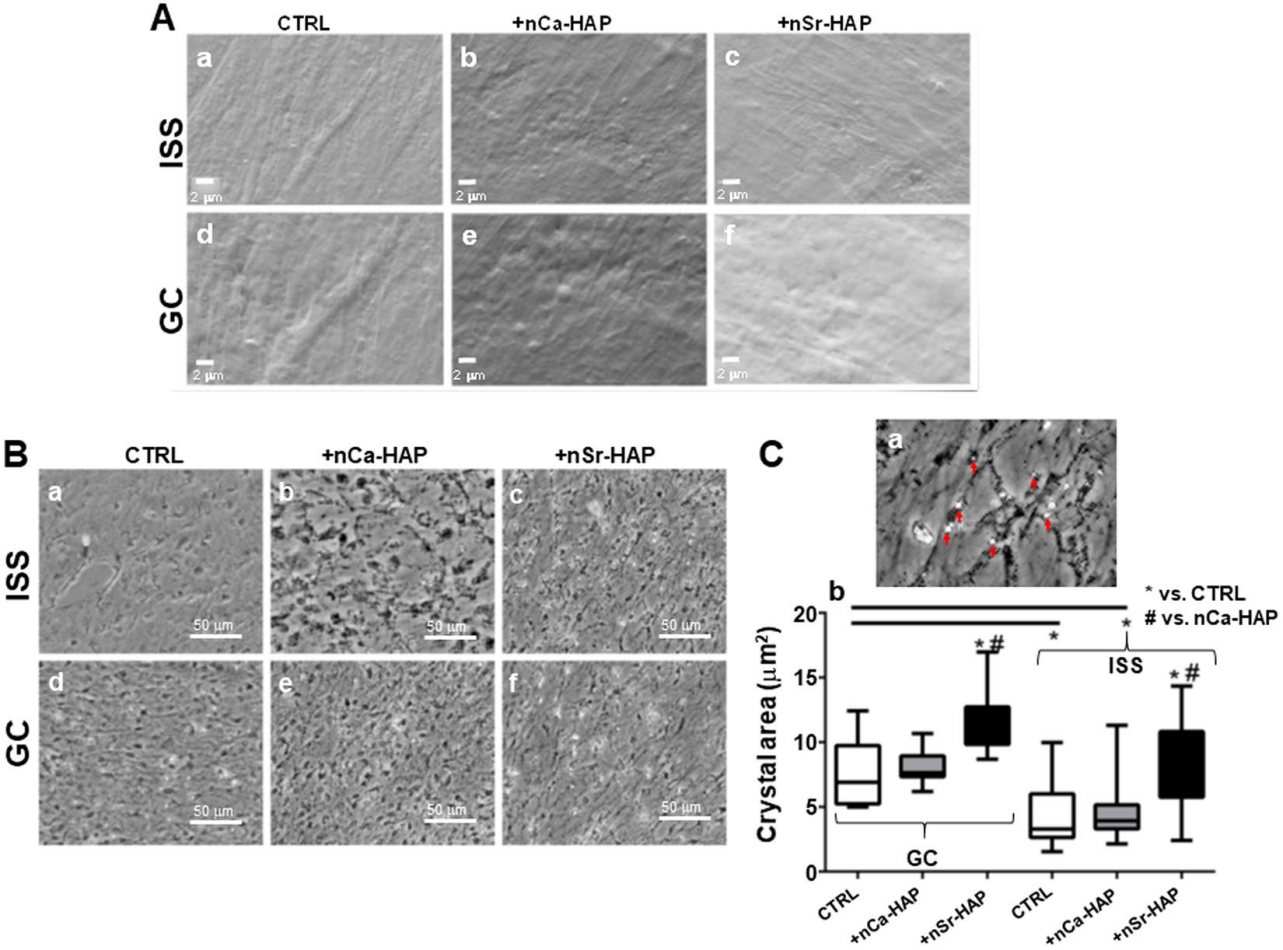
Space flight experiment features. (**A**) Representative SEM images of hBM-MSCs seeded on Thermanox coverslips before the ISS Space Flight and Ground Control experiments: untreated cells (panels **a** and **d**), nCa-Hap (panels **b** and **e**) and nSr-HAP (panels **c** and **f**) treated cells. Magnification 5.000X, scale bars represent 2 μm. (**B**) Phase-contrast microscope imaging of Thermanox recovered from both GC (panels **d–f**) and ISS (panels **a–c**) EUs. (**C**) panel **a**, crystal imaging for quantitative analysis of their size in flown hBM-MSCs treated with nSr-HAP; deposited crystals are shown by red arrows as an example; panel **b**, crystal size quantitative analysis of images. Tree images for each thermanox were evaluated, measuring at least 20 crystals. Statistical significance values are indicated as *p < 0.05 vs CTRL, and ^#^p < 0.05 vs nCa-HAP RPM.

The preparatory operations for the space flight experiment started with the seeding of hBM-MSCs on 15 Thermanox slides. Twelve EUs out of 15 were destined for the space flight experiment, while the others were used for the evaluation of cell viability and proliferation (data not shown) and finally fixed for SEM observations (Fig. 8A). The cells were cultured in PM conditions and this yielded a 70% increase in cell number over a three-day period before the launch, reaching the required number of cells to be used for the ISS experiment. These cells, used for the experiment onboard the ISS, were inserted in six STROMA EUs (the EU numbers assigned to treatments were: #124 and #125, CTRL; #126 and #127, +nCa-HAP; # 128 and #129, +nSr-HAP) assembled and integrated into the KICs, and stored at 37 °C until their transfer to a BIOKIT (Table 1). The BIOKIT is made of certified materials with good insulation and thermal properties. BIOKITs have predominantly been used to support flight HW transportation since their first use in 2003, for the STROMA mission loading on Dragon X (the orbital transport capsule for the material destined for the ISS). The space launch of the samples took place on April 14^th^, 2015, in the context of the sixth SpaceX commercial space mission CRS-6. The transfer of samples to the ISS took place two days later, while the beginning of the in-flight experiment started on April 18^th^ 2015 with the loading of samples into the KUBIK incubator. When the KICs were inserted in the KUBIK the first medium change from reservoir #2 took place. Cells were cultured for 88 hours and then washed two times with PBS from reservoirs #3 and #4 and, finally, the RNA Cell Protector was inserted in the culture chamber to stabilize the RNA samples (Fig. 6, panel d). The pistons of the reservoirs were activated in series, allowing for medium changes. Cells fixed by RNA cell protector injection, were stored on board ISS initially at 4 °C. The KICs were then transferred at −95 °C into the MELFI until the return to Earth and handed over to our lab. Once back from the ISS, data relating to times and temperatures occurring during the experiment on board the ISS were recovered to allow for the reproduction of the entire experiment (Fig. S4). Temperature recordings showed that the temperature never dropped below 26 °C during upload and that the incubation in the KUBIK was carried out at 37 °C as requested (Fig. S4, panel a). At the end of the incubation period, the temperature of the samples frozen in MELFI were less than −96 °C (Fig. S4, panel b).

**Table 1:** NATO experiment of the FUTURA Space Mission onboard the ISS.

NATO Payload Experiment Table Summary				
**Experiment**	**Institutions involved**	**# STROMA EU** **ISS GC**		**Facility EC**
NATO	-University of Pavia (PI) -University of Milano (Co-PI) -Institute of Crystallography-CNR (Co-PI) -Kaiser Italia Srl (Co-PI)	#124, #125, #126, #127, #128, #129	#116, #117, #118, #119, #120, #121	1 BIOKIT
**Experiment duration**		**Experiments temperature profiles**		**Suitable temperature control facilities**
Max. 3 days		● Incubation: 37 °C ● Upload:23 °C ≤ T ≤ 30 °C ● Storage:T ≤ −20 °C ● Retrieval:T ≤ −20 °C Temperature recordings by i-button data logger		● MERLIN, BIOKIT ○ (Upload:23 °C ≤ T ≤ 30 °C) ● MELFI, MERLIN ○ (Storage: T ≤ −20 °C) ● COLDBAG with ICEPAC’s ○ (Download ≤ −20 °C)

^#^Indicates the identification number of each STROMA EU used on ISS and GC.

Unfortunately, after ISS experiment, both EUs #124 and #126 evidenced pronounced levels of contamination. Nevertheless, the RNA cell protector was recovered from all of the culture chambers and RNAs and proteins were isolated. The quality of RNA was evaluated by Agilent BioAnalyzer 2100. The results, reported in Fig. S5, indicate that 4 out of 6 samples had a respectable level of RNA quality (with RNA integrity numbers between 7.5 and 8.6). The RNA samples extracted from the two contaminated EUs were completely degraded. This contamination means that it is not possible to obtain statistically significant results from any RNA Sequence analysis and, as a result, has limited our ability to test the effects of nCa-HAP and nSr-HAP on hBM-MSCs cell differentiation in real microgravity conditions, onboard of the ISS. No study of RNA Seq were performed on GC.

Interestingly, after the removal of the samples for the RNA Seq analysis, the Thermanox coverslips were immediately recovered and observed under the microscope (Fig. 8B). The images of the recovered Thermanox of both the ISS (Fig. 8B, panels a–c) and GC (Fig. 8B, panels d–f) samples and the results extrapolated by crystal size quantitative analysis (Fig. 8C, panels b) are also reported. The crystals of the ISS’s untreated samples were smaller than those observed in GC conditions (p < 0.05) (Fig. 8C, panels b). In both culture conditions, ISS and GC, we observed an increment in crystal dimension for nSr-HAP treated samples when compared with nCa-HAP and untreated samples (p < 0.05) (Fig. 8C, panels b). It appears that nSr-HAP exerts a positive effect on extracellular matrix mineralization even in absence of gravity, as already demonstrated in 1 g condition (Fig. 3).

Collectively, these data, even if preliminary, shed a light on possibilities to mediate the physiological response of osteoblasts to nanoparticle treatments, allowing us to set up new on-ground experiments to unravel the molecular mechanisms underlying the osteoblast’s activity.

## Conclusions and Future Perspectives

In our current work, stable suspensions of nSr-HAP were systematically synthesized and used to test their effects on hBM-MSCs cells during differentiation. These suspensions exhibited high levels of biocompatibility. This is confirmed by viability results and cell morphology evolution as well as a demonstrated capability to promote cell adhesion and proliferation. *In vitro*, these positive effects were enhanced by prolonged incubation periods and high Sr concentrations that induced cell apoptosis or death.

We observed an increased expression of bone marker genes during the differentiation phase, along with enhanced levels of mineralization and calcified matrix deposition in nSr-HAP-treated cells. This clearly indicates these nanoparticles have a role in driving a more rapid commitment of stem cells toward osteoblastic differentiation. In simulated microgravity conditions, the nSr-HAP treated cells were able to counteract microgravity-induced ALP reduction and, on ISS sample, stimulated the dimensions of crystals during hBM-MSCs differentiation. It was, unfortunately, not possible to test transcriptomic effects in real space condition due to sample contamination. Further research is now needed to understand the molecular mechanisms activated by nSr-HAP nanoparticles. The effect of both nCa-Hap and nSr-HAP suspensions on osteoclasts and osteocytes *in vitro* cultures is under investigation and work has been planned on osteoblast-osteoclast co-cultures. Moreover, taking into account the relevance of the Wnt/β-catenin pathways in skeletal biology and disease, additional studies have been planned in order to test the possible involvement of nCa-Hap, nSr-HAP and/or Sr^2+^ in this signalling pathway, either in osteoblasts or in osteoclasts.

We may state, however, that the results achieved in 1 g and in simulated microgravity conditions clearly suggest that nSr-HAP could be used to delivery Sr^2+^ to bone tissue and promote regeneration, as a component of a synthetic bone substitute material, via pharmaceutical preparations or as food supplements for systemic distribution.

## Materials and Methods

### nCa-HAP and nSr-HAP synthesis, suspension preparation and chemical-physical characterization

The nCa-HAP and nSr-HAP were synthetized, suspended and characterized as previously reported^19^.

### Cell Culture

#### 1 g conditions

The Institutional Review Board of the University of Pavia (2011) approved this study. All methods were performed in accordance with the relevant guidelines and regulations. Informed consent was obtained from all participants and their legal guardians. The hBM-MSC samples were isolated and phenotypically analysed to assess their mesenchymal properties according to the International Society for Cellular Therapy, as previously described^34^. As reported in our previous studies^35,36^, hBM-MSCs were cultured at 37 °C in a humidified incubator with 5% CO_2_ in a medium maintenance, low-glucose DMEM (Dulbecco’s modified Eagle’s medium) supplemented with 10% FBS, 1% glutamine, 50 μg/ml penicillin-streptomycin (P-S) and amphotericin B (Lonza Group Ltd.) (Proliferative medium, PM). hBM-MSCs were cultured in osteogenic medium (OM), α-MEM (Minimum Essential Medium) supplemented with 10% FBS, 50 μg/ml of P-S and the osteogenic mixture containing 100 nM dexamethasone, 5 mM β-glycerophosphate disodium and 50 mg/ml ascorbic acid (Sigma-Aldrich, S. Louis, MO, USA) to induce osteogenesis. The treatment lasted up to 28 days and the medium was changed every 3 days: for the samples cultured in PM or OM conditions the media was replaced with fresh culture media with/without nCa-HAP or nSr-HAP.

#### Random positioning machine (RPM)

Microgravity conditions were simulated using the RPM (Dutch Space, Leiden, Netherlands)^37^. The RPM provides continuous random changes in orientation of the 1 g vector for an accommodated experiment. For the EST experiment, 150 × 10^3^ hBM-MSCs were seeded on Thermanox supports (11 × 22 mm) and cultured in PM for 72 h before the samples were inserted into the STROMA EUs and then exposed to the RPM conditions. The STROMA EUs were supplemented with 12.5 mM HEPES (Sigma-Aldrich) to remove air bubbles and fixed on the RPM as close as possible to the centre of the platform, which was then rotated using the real random mode (random speed and random direction) of the machine. The RPM operated at 37 °C for 88 h, mirroring the duration of the ISS NATO space flight experiment. During the RPM experiment the samples cultured in OM, with and without nCa-HAP or Sr-HAP, were unexposed (Ground Control, GC) or exposed to RPM. The GC cultures, treated in parallel in identical equipment, were then placed on the RPM.

### Cell viability

The testing of samples cultured in OM, both with and without nCa-HAP or nSr-HAP, was performed in 1 g and RPM conditions and cell mitochondrial activity was evaluated using 3-(4,5-dimethylthiazole-2-yl)-2,5-diphenyl tetrazolium bromide (MTT) test (Sigma-Aldrich) at 1, 3, 7, 8 and 28 days, as previously reported^38^. Aliquots of 100 μL were sampled and their absorbance was measured at reference wavelengths of 595 nm and 650 nm by a microplate reader (BioRad Laboratories, Hercules, CA, USA).

### Immunofluorescence studies

#### Apoptosi

The hBM-MSCs were labelled using the PSVue480™ cell stain, according to the manufacturer’s instructions (Molecular Targeting Technologies, Inc.), in order to determine the extent to which cell death resulted from the addition of the nanoparticle suspension from the culture medium. PSVue480™ dye detects apoptosis by targeting the loss of phospholipid asymmetry in the plasma membrane, an early event in the apoptosis process irrespective of cell type, which results in the exposure of phosphatidylserine (PS) residues at the outer plasma membrane leaflet. The hBM-MSCs were seeded on glass coverslips (Thermo Scientific) with a density of 3 × 10^4^ cells/cm^2^ and incubated with H_2_O_2_ (positive control; 100 mM), no addition (negative control) and with 62.5 μg/mL concentrations of nCa-HAP or nSr-HAP for 24 h. At the end of each culture, cells were stained with PSVue480™ solution. Samples were then counterstained with a Hoechst 33342 solution (2 μg/mL) to target the cellular nuclei and then observed under a fluorescence optical microscope (Nikon Eclipse 80i).

#### Morphological studies

The hBM-MSCs which were cultured in PM culture conditions for 24 h, either with or without the 62.5 μg/mL concentration of nCa-HAP or nSr-HAP, were fixed with a 4% (w/v) paraformaldehyde (PFA) solution in a 0.1 M phosphate buffer (pH 7.4) for 30 min at 4 °C and then washed with PBS three times. The hBM-MSCs were then made permeable with a 0.1% Triton X-100 solution for 1 h at room temperature (RT) and incubated with FITC conjugated antibody against β-tubulin (Invitrogen) for 90 min at RT. F-actin staining was performed using 10 μg/mL phalloidin, labelled with tetramethylrhodamine (TRITC) (Sigma) for 45 min. Lastly, nuclei were counterstained with Hoechst 33342 solution (2 μg/mL). Samples were examined using a Confocal Laser Scanning Microscope (CLSM) (Leica TCS SP2, Leica Instruments, Germany), acquiring images every 1.5 μm to a depth of 100 μm.

#### Bone extracellular matrix deposits

The hBM-MSCs cultured in OM culture conditions for 28 days, with or without the 62.5 μg/mL concentrations of nCa-HAP or nSr-HAP, were fixed with a 4% (w/v) paraformaldehyde solution in 0.1 M phosphate buffer (pH 7.4) for 30 min at 4 °C and washed with phosphate-buffered saline (PBS) three times. The cells were then blocked by incubating with PAT (PBS containing 1% [w/v] bovine serum albumin and 0.02% [v/v] Tween 20) for 2 h at room temperature and then washed. Cells were stained with anti-type-I collagen, anti-osteocalcin and anti-alkaline phosphatase (diluted to 1:500 in PAT) overnight at 4 °C. This was followed by an incubation with an Alexa-Fluor-488 goat anti-rabbit IgG (HþL; Invitrogen) at a dilution of 1:750 in PAT for 1 h at room temperature. At the end of the incubation period the samples were washed in PBS, counterstained with a Hoechst solution (2 μg/mL) to target the cellular nuclei and then washed. The images were taken by the Fluorescence microscope (Leica Microsystems, Bensheim, Germany) equipped with a digital image capture system at 40X magnification.

### Alkaline phosphatase (ALP) activity

The ALP activity of the hBM-MSCs, cultured in PM or OM culture conditions with or without the nCa-HAP or nSr-HAP suspensions, was determined using a colorimetric end point assay as previously described^39^. The assay measures the conversion of the colourless substrate *p*-nitrophenol phosphate (PNPP) by the enzyme ALP to the yellow product *p*-nitrophenol, in this process the rate of the colour change corresponds to the amount of enzyme present in the solution. In short, an aliquot (1 mL) of 0.3 M PNPP (dissolved in glycine buffer, pH 10.5) was added to each scaffold at 37 °C. After incubation, the reaction was stopped by the addition of 100 μL 5 M NaOH. The standards of PNPP, in concentrations ranging from 0 to 50 μM, were freshly prepared from dilutions of a 500 μM stock solution and incubated for 10 min with 7U of ALP (Sigma-Aldrich), previously dissolved in 500 μL of ddH_2_O. The absorbance reading was performed at 405 nm with a microplate reader (BioRad Laboratories) using 100 μL of standard or sample placed into individual wells on a 96-well plate. Samples were run in triplicate and compared against a calibration curve of p-nitrophenol standards. The enzyme activity was expressed as micromoles of *p*-nitrophenol produced per minute per milligram of enzyme (for analysis in 1 g culture conditions) and was normalized in relation to cell number seeded in the plate for analysis in STROAM EU on RPM culture conditions x 88 h.

### Alizarin red staining

After 28 days, the hBM-MSCs cultured in PM or OM culture conditions, both the untreated samples and those treated with a 62.5 μg/mL concentration of nCa-HAP or nSr-HAP, were rinsed with PBS, fixed for 30 min at 4 °C with 4% PFA and stained for 10 min with 40 mM of Alizarin Red S (pH 4.2, Sigma-Aldrich) to analyse the Ca deposits. Samples were then washed three times with water and once with PBS for 10 min and observed under the microscope. Alizarin Red S staining was released from the cell matrix by incubation in a 10% cetylpyridinium chloride (Sigma-Aldrich) solution in 10 mM of sodium phosphate (pH 7.0) for 15 min and the absorbance was measured at 562 nm.

### Gene expression

After 28 days, we extracted RNA from hBM-MSCs that have been cultured in PM or OM culture conditions and either untreated or treated with a 62.5 μg/mL solution of nCa-HAP or nSr-HAP. This was performed with the RNeasy Plus Mini Kit (Qiagen) and retro-transcribed into cDNA with the iScript cDNA Synthesis Kit (BioRad Laboratories), as previously reported^40^. A quantitative reverse-transcription polymerase chain reaction (qRT-PCR) analysis was performed in a 48-well optical reaction plate using a MiniOpticon Real-Time PCR System (BioRad Laboratories). The oligonucleotide primers were designed with gene sequences published in GenBank, indicated in Table S2. Reactions were performed in 20 μL with 2 μL of cDNA, 10 μL Brilliant SYBER Green qPCR Master Mix (Stratagene, La Jolla, CA), 0.4 μL of each primer, and 7.2 μL H_2_O. The PCR conditions were as follows: 3 min at 95 °C, 40 cycles of 5 sec at 95 °C, and 23 sec at 60 °C. Gene expression was normalized to the 18 S housekeeping gene expression. Each sample was analysed in triplicate and correlated against a standard curve. The reaction mixture, without cDNA, was used as a negative control in each run.

The same analysis was performed on samples cultured in the STROMA EU and exposed to RPM for 88 h.

### Extraction of ECM proteins and enzyme-linked immunosorbent assay

After 28 days, the untreated hBM-MSC samples and those treated with a 62.5 μg/mL solution of nCa-HAP or nSr-HAP, in PM or OM culture conditions, were incubated in a sterile lysis buffer composed of 20 mM Tris-HCl, 4 M GuHCl, 10mMEDTA, 0.066% [w/v] sodium dodecyl sulphate (SDS), at pH 8.0, and frozen and thawed several times. After that, the total protein concentration of all samples was evaluated with the BCA Protein Assay Kit (Pierce Biotechnology, Inc., Rockford, IL). At this point we completed the calibration curves to measure type-I and -III collagens, decorin, osteopontin, osteocalcin, osteonectin, fibronectin and anti-alkaline phosphatase. The Microtiter wells were coated with increasing concentrations of purified protein, from 10 ng to 2 μg, in a coating buffer (50 mM Na_2_CO_3_, pH 9.5) overnight at 4 °C. Control wells were coated with bovine serum albumin (BSA) as a negative control. Microtiter wells were coated, overnight at 4 °C, with 100 μL of the previously extracted ECM (20 μg/mL in coating buffer) in order to measure the ECM amount of each protein by ELISA. After three washes with PBS containing 0.1% (v/v) Tween 20, the wells were blocked by incubating them with 200 μL of PBS containing 2% (w/v) BSA for 2 h at 22 °C. The wells were subsequently incubated for 1.5 h at 22 °C with 100 μL of the anti-type-I and -III collagens, antidecorin, anti-osteopontin, anti-osteocalcin, anti-osteonectin and anti-alkaline phosphatase rabbit polyclonal antisera (1:500 dilution in 1% BSA), kindly provided by L. Fisher. The same dilution was used for the anti-fibronectin rabbit polyclonal IgG. After washing, the wells were incubated for 1 h at 22 °C with 100 μL of horseradish peroxidase (HRP) and conjugated goat anti-rabbit IgG (1:1000 dilution in 1% BSA). The wells were finally incubated with 100 μL of the development solution (phosphate-citrate buffer with o-phenylenediamine dihydrochloride substrate). The colour reaction was stopped with 100 μL of 0.5 M H_2_SO_4_, and the absorbance values were measured at 490 nm with a microplate reader (BioRad Laboratories).

### Experiment performed on ISS

This work was performed within the context of a Memorandum of Understanding, since 2001, between the Italian Space Agency (ASI) and NASA which allows for the scientific utilization of the International Space Station (ISS) and has provided ASI with exploitation rights. As a result of this agreement, ASI has conducted a number of experiments on board the ISS, mostly in the field of Life Sciences and technological development.

The ESA astronaut selected to conduct the NATO project experiments was Samantha Cristoforetti, on board of the ISS during her mission entitled **FUTURA**. She reached the ISS on-board of the Soyuz TMA-15 Russian capsule that was launched on November 23^rd^, 2014 and returned to Earth on June 15^th^, 2015.

A summary of the NATO experiment conducted onboard the ISS, one of the nine scientific research and technology projects selected for this mission by ASI, is reported in Table 1. NATO was designed to evaluate the influence of *hydroxyapatite nanoparticles enriched with or without strontium* on osteoblasts differentiation in real space environment by using the RNA Seq techniques^41,42^.

The hBM-MSCs were cultured in 6 STROMA EUs and then inserted in 6 KICs, and housed inside the KUBIK incubator for 3 days onboard of the ISS. The NATO experiment was prepared during the first two weeks of April 2015 at the Space Kennedy Center laboratories in Cape Canaveral (Florida, USA) before being transferred on the SpaceX CRS-6 cargo ship to the ISS. Six EUs were fully automated and managed by an internal microcomputer for the experiment onboard of the ISS, the other 6 EUs for GC were in a manual setting.

The STROMA experimental units (EU) provided by Kayser Italia (Kayser Italia srl, http://www.kayser.it/) were used to cultivate cells in with and without nCa-HAP and nSr-HAP nanoparticles in order to study the effect of these nanoparticles on the levels of hBM-MSC differentiation to osteoblasts on the ISS: 6 STROMA EU were launched on ISS and 6 were cultured on Ground. 3 × 10^5^ cells were seeded on 12 Thermanox coverslips (Nunc) and left to attach for 48 h. In addition, the medium was replaced with OM with and without nCa-HAP or nSr-HAP nanoparticles at the same concentration. Cell viability was examined with the Alamar Blu assay (Sigma-Aldrich), according to the manufacturer’s instructions. After 8 days, but before the ISS experiments, a few samples were fixed for SEM observations, as previously described^39,40^. For the other samples, media were collected and kept at −80 °C and the Thermanox were built inside the culture chamber of the STROMA EU with a fresh medium with and without nanoparticles plus the addition of 12.5 mM HEPES (Sigma).

#### Filling the EU Reservoirs

Four of the five EU reservoirs were filled as follows: #2 with a fresh medium with or without nanoparticles (depending of the EU), #3 PBS (first washing), #4 PBS (second washing) and #5 RNA Cell Protector (Qiagen). 6 EUs inside 6 KICs (Kayser Italia srl, http://www.kayser.it/) were shipped inside a BIOKIT, ensuring a constant temperature of 37 °C during their transportation to the ISS through the space vector Falcon 9 of the SpaceX CRS6, launched from SLC-40 (Space Launch Complex 40) on April 14^th^, 2015 from Kennedy Space Center – Cape Canaveral – Florida (USA). The payload reached the ISS on April 17^th^, 2015 and was docked by Captain Samantha Cristoforetti who inserted the KICs inside the KUBIK, the incubator present on ISS. When the KICs were inserted in the KUBIK, the first medium change from reservoir #2 took place. Cells were cultivated for 88 hours and then washed two times with PBS from reservoirs #3 and #4 and, finally, the RNA Cell Protector was inserted into the culture chamber to stabilize RNA. The EUs were then transferred by Captain Samantha Cristoforetti from the KUBIC to the −95 °C MELFI. The 6 KICs of GC followed the same procedures, but manually performed. On May 21^th^, 2015 the 6 ISS KICs frozen samples were carried to Earth by Dragon 6 vector, splashing down in the Pacific Ocean at 12.58 pm. The samples were then shipped to Italy.

As with the GC samples, the 6 manual EUs were assembled on the same day as the 6 EUs destined for the experiment on board of the ISS. The 6 GC EUs were kept at a temperature of 25 °C and sent to Italy. At this point they were transferred to an incubator at 26–28 °C until the beginning of the space flight experiment on board ISS, to simulate sample handling and temperature before ISS experiment. Eventually, the samples were activated and incubated at 37 °C for 88 h according to the flight experiment scheme, and at the end of incubation were stored at −80 °C until their opening with the flight samples.

#### ISS and GC samples analysis

The 12 KICs (6 ISS and 6 GC) were opened on June 9^th^, 2015 and the RNA cell protector from the culture chambers, the Thermanox and culture media from washings were recovered. RNA (Fig. S4) and proteins (Table S3, the subject of on-going studies) were extracted from RNA cell protector using RNeasy Mini Kit (Qiagen), according to the manufacturer’s instructions, and stored at −80 °C.

RNA was qualitatively analysed by Agilent BioAnalyzer 2100 in order to perform RNA Seq.

For morphological analyses of deposited hydroxyapatite crystals, images of samples were acquired by a Nikon Eclipse Te200 equipped with 20x magnification and a Nikon Ds-Fi1 camera (sense: 5 MP CMOS) controlled by a Ds-U3camera controller and saved in TIFF format. Image analyses were performed under pre-calibrated ImageJ and the deposited hydroxyapatite crystals were manually segmented one by one in order to obtain crystal area.

### Statistical analysis

Quantitative results are expressed as the mean ± standard error of the mean (SEM). One-way analysis of variance (ANOVA) with post hoc Bonferroni test was applied, with a significance level of 0.05 in order to compare results. For crystal images of deposited crystals, statistical analysis was performed by 1 Way ANOVA Holm-Sidak’s multiple comparisons test (Graph Pad Software in., San Diego, USA) and p value < 0.05 was considered statistically different.

## Supplementary information

Supplementary Info

## Data Availability

The datasets generated during and/or analysed in the current study are included in this published article (and its Supplementary Information file).

## References

[1] Raisz LG (2005). Pathogenesis of osteoporosis: concepts, conflicts, and prospects. J. Clin. Invest..

[2] Alexandre C, Vico L (2011). Pathophysiology of bone loss in disuse osteoporosis. Joint Bone Spine.

[3] Rodan GA, Martin TJ (2000). Therapeutic approaches to bone diseases. Science.

[4] South-Paul JE (2001). Osteoporosis: part I. Evaluation and assessment. Am. Fam. Physician.

[5] Moshiri A, Sharifi AM, Oryan A (2017). Current Knowledge, Drug-Based Therapeutic Options and Future Directions in Managing Osteoporosis. Clin. Rev. Bone Miner. Metab..

[6] Dahl SG (2001). Incorporation and distribution of strontium in bone. Bone.

[7] Marie PJ (2003). Optimizing bone metabolism in osteoporosis: insight into the pharmacologic profile of strontium ranelate. Osteoporos. Int..

[8] Reginster JY (2015). The position of strontium ranelate in today’s management of osteoporosis. Osteoporos. Int..

[9] Fricain JC (2013). A nano-hydroxyapatite–pullulan/dextran polysaccharide composite macroporous material for bone tissue engineering. Biomaterials.

[10] Narayanan G, Vernekar VN, Kuyinu EL, Laurencin CT (2016). Poly (lactic acid)-based biomaterials for orthopaedic regenerative engineering. Adv Drug Deliver Rev.

[11] Yin P, Feng F, Lei T, Jian X (2012). Colloidal-sol gel derived biphasic FHA/SrHA coatings. Surf. Coatings Technol..

[12] Gentleman E (2010). The effects of strontium-substituted bioactive glasses on osteoblasts and osteoclasts *in vitro*. Biomaterials.

[13] Ni GX (2006). Strontium-containing hydroxyapatite (Sr-HA) bioactive cement for primary hip replacement: an *in vivo* study. J. Biomed. Mater. Res. B. Appl. Biomater..

[14] Aina V (2013). Sr-containing hydroxyapatite: morphologies of HA crystals and bioactivity on osteoblast cells. Mater. Sci. Eng. C. Mater. Biol. Appl..

[15] Abdel-Aal EA (2011). Inserting of strontium during coating of hydroxyapatite compound on titanium substrate. Int. J. Nanoparticles.

[16] Raucci MG, Giugliano D, Alvarez-Perez MA, Ambrosio L (2015). Effects on growth and osteogenic differentiation of mesenchymal stem cells by the strontium-added sol–gel hydroxyapatite gel materials. J. Mater. Sci. Mater. Med..

[17] Kitayama S (2015). Regeneration of rabbit calvarial defects using biphasic calcium phosphate and a strontium hydroxyapatite-containing collagen membrane. Clin. Oral Implants Res..

[18] Capuccini C (2009). Interaction of Sr-doped hydroxyapatite nanocrystals with osteoclast and osteoblast-like cells. J. Biomed. Mater. Res. A.

[19] Frasnelli M (2017). Synthesis and characterization of strontium-substituted hydroxyapatite nanoparticles for bone regeneration. Materials Science and Engineering: C.

[20] Campi G (2017). Heterogeneous and self-organizing mineralization of bone matrix promoted by hydroxyapatite nanoparticles. Nanoscale.

[21] Collet PH (1997). Effects of 1-and 6-month spaceflight on bone mass and biochemistry in two humans. Bone.

[22] Vico L (2000). Effects of long-term microgravity exposure on cancellous and cortical weight bearing bones of cosmonauts. The Lancet.

[23] Grimm D (2016). The impact of microgravity on bone in humans. Bone.

[24] Tamma R (2009). Microgravity during spaceflight directly affects *in vitro* osteoclastogenesis and bone resorption. FASEB J..

[25] Ulbrich C (2014). The Impact of Simulated and Real Microgravity on Bone Cells and Mesenchymal Stem Cells. Biomed Res Int.

[26] Lang TF, Leblanc AD, Evans HJ, Lu Y (2006). Adaptation of the proximal femur to skeletal reloading after long‐duration spaceflight. J Bone Miner Res..

[27] Komori T (1997). Targeted Disruption of Cbfa1 Results in a Complete Lack of Bone Formation owing to Maturational Arrest of Osteoblasts. Cell.

[28] Campi G (2015). Imaging collagen packing dynamics during mineralization of engineered bone tissue. Acta biomaterialia.

[29] Campi G (2013). Imaging regenerating bone tissue based on neural networks applied to micro-diffraction measurements. Applied Physics Letters.

[30] van Dijk S, D’Errico JA, Somerman MJ, Farach-Carson MC, Butler WT (1993). Evidence that a non-RGD domain in rat osteopontin is involved in cell attachment. J. Bone Miner. Res..

[31] Grzesik WJ, Robey PG (1994). Bone matrix RGD glycoproteins: immunolocalization and interaction with human primary osteoblastic bone cells *in vitro*. J. Bone Miner. Res..

[32] Genchi GG (2018). Modulation of gene expression in rat muscle cells following treatment with nanoceria in different gravity regimes. Nanomedicine (Lond)..

[33] Borst A, van Loon JJ (2009). Technology and development for the random positioning machine, RPM. Microgravity Sci Technol..

[34] Bernardo ME (2007). Optimization of *in vitro* expansion of human multipotent mesenchymal stromal cells for cell-therapy approaches: further insights in the search for a fetal calf serum substitute. J Cell Physiol..

[35] Bloise N (2018). The effect of pulsed electromagnetic field exposure on osteoinduction of human mesenchymal stem cells cultured on nano-TiO2 surfaces. PLoS One.

[36] Vercellino M (2016). Nanostructured TiO_2_ Surfaces Promote Human Bone Marrow Mesenchymal Stem Cells Differentiation to Osteoblasts. Nanomaterials (Basel).

[37] Benavides Damm T, Walther I, Wüest SL, Sekler J, Egli M (2014). Cell cultivation under different gravitational loads using a novel random positioning incubator. Biotechnol. Bioeng..

[38] Saino E (2010). *In Vitro* Enhancement of SAOS-2 Cell Calcified Matrix Deposition onto Radio Frequency Magnetron Sputtered Bioglass-Coated Titanium Scaffolds. Tissue Eng. Part A.

[39] Bloise N (2018). Ether-Oxygen Containing Electrospun Microfibrous and Sub-Microfibrous Scaffolds Based on Poly(butylene 1,4-cyclohexanedicarboxylate) for Skeletal Muscle Tissue Engineering. Int J Mol Sci..

[40] Cristofaro F (2018). Influence of the nanofiber chemistry and orientation of biodegradable poly(butylene succinate)-based scaffolds on osteoblast differentiation for bone tissue regeneration. Nanoscale.

[41] Stark Rory, Grzelak Marta, Hadfield James (2019). RNA sequencing: the teenage years. Nature Reviews Genetics.

[42] Li L (2019). Effects of simulated microgravity on the expression profiles of RNA during osteogenic differentiation of human bone marrow mesenchymal stem cells. Cell Prolif..

