# Supplementary material for: The NATO project: nanoparticle-based countermeasures for microgravity-induced osteoporosis
Source: Sci Rep. 2019 Nov 20;9:17141. doi: 10.1038/s41598-019-53481-y (PMC6868153; doi:10.1038/s41598-019-53481-y)
Supplement: Supplementary file 1 — Supplementary Info [file 41598_2019_53481_MOESM1_ESM.docx]

**Supplementary Information**

***The NATO project: nanoparticle-based countermeasures for microgravity-induced osteoporosis***

F. Cristofaro^a,b,°^, G. Pani^c°^, B. Pascucci^d^, A. Mariani^e^, M. Balsamo^e^, A. Donati^e^, G. Mascetti^f^, G. Rea*^d^, A.M. Rizzo*^c^, L. Visai*^a,b^

^a^Molecular Medicine Department (DMM), Center for Health Technologies (CHT), UdR INSTM, University of Pavia, Viale Taramelli 3/B – 27100 Pavia – ITALY

^b^Department of Occupational Medicine, Toxicology and Environmental Risks, Istituti Clinici Scientifici Maugeri S.p.A, IRCCS, Via S. Boezio, 28 - 27100 Pavia – ITALY

^c^Department of Pharmacological and Biomolecular Sciences, Università degli Studi di Milano, via D. Trentacoste 2, 20134 Milano, ITALY.

^d^Institute of Crystallography - CNR, via Salaria Km 29.300, 00015, Monterotondo Roma, ITALY

^e^ Kayser Italia, S.r.l., Via di Popogna, 501 - 57128 Livorno - ITALY

^f^ Italian Space Agency, ASI, Via del Politecnico, 00133 Roma, ITALY

°Both authors equally contributed to this article

***Corresponding authors:**

[livia.visai@unipv.it](mailto:livia.visai@unipv.it), [angelamaria.rizzo@unimi.it](mailto:angelamaria.rizzo@unimi.it), [giuseppina.rea@ic.cnr.it](mailto:giuseppina.rea@ic.cnr.it)

**Figure 1S**. **Representative images of untreated and treated hBM-MSCs with both types of nanoparticle suspensions**. The treated samples were cultured for 24h (**panels a-c**) and 72h (**panels d-f**) either without (**panels a and d**) or with 62.5 μg/mL nCa-HAP (**panels b and e**) or nSr-HAP (**c and f**) solutions and observed throughout Optical Microscope (**panels a-f**) or SEM (**panels g-i**) at 72h. The scale bar represents 20μm per Optical Microscopy images and 2μm for SEM observations.


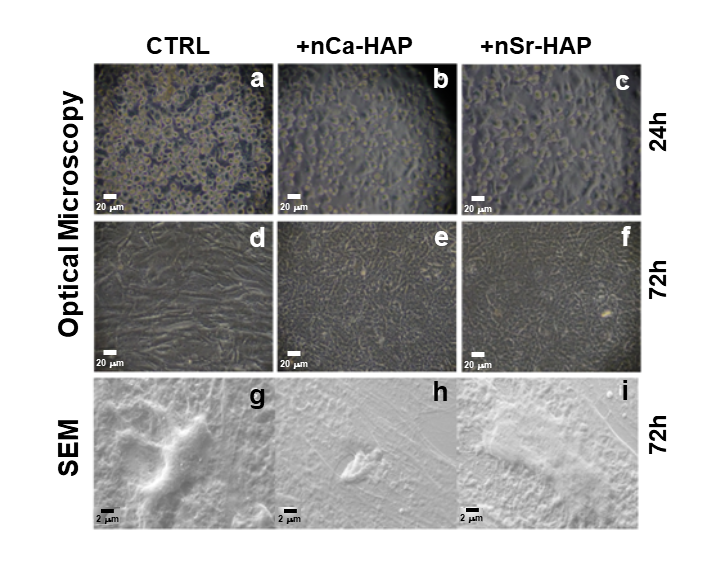


**Figure 2S**. **Dose-dependent ALP activity of nCa-HAP or nSr-HAP treated hBM-MSCs for 28 days in both PM and OM conditions**. Results are expressed as percentage related to the untreated cells. The data are presented as the average ± standard deviations for three measurements in two separate experiments. Statistical significance values are indicated as **p<0.01, and ***p<0.001.


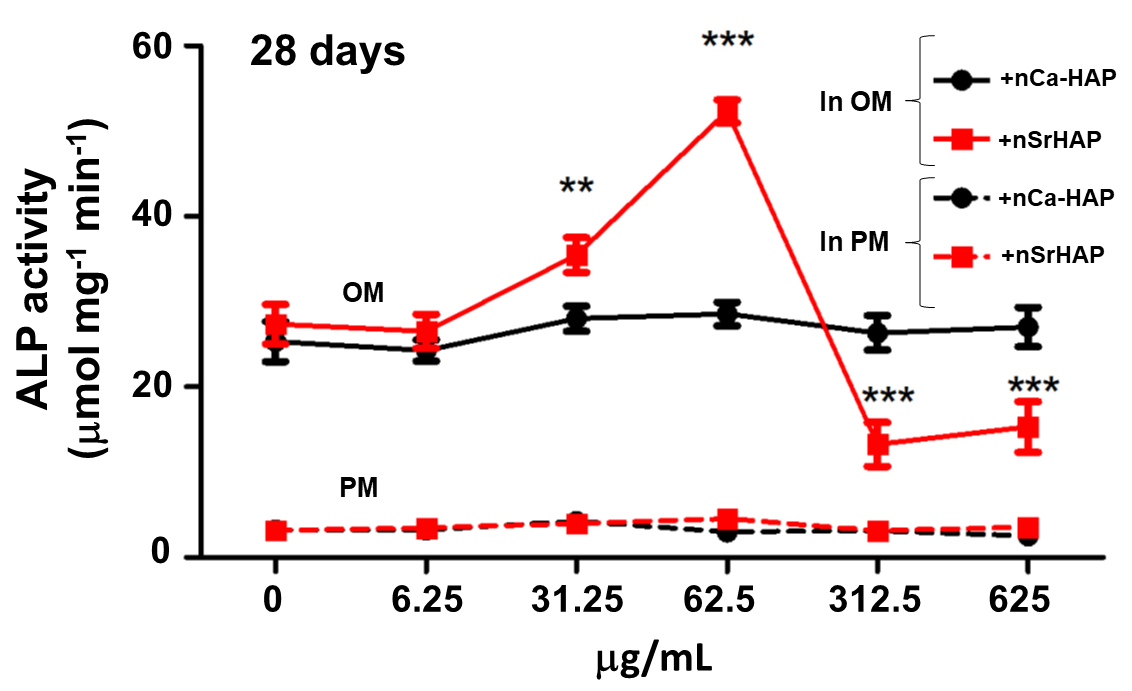


**Figure 3S**. **Effect of simulated microgravity on gene marker expression of hBM-MSCs cultured in STROMA EU.** The hBM-MSCs were cultured in OM conditions for 3 days in simulated microgravity (RPM) and in 1g conditions as a control (GC). The *Runx-2*, *BOSP*, *COL 1* and *ALP* gene expression are presented as the average ± standard deviations for three measurements in two separate experiments. Statistically significance values are indicated as **p<0.01, and ***p<0.001.


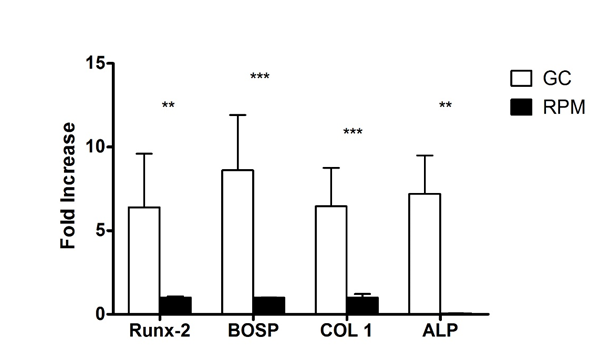


**Figure 4S.** **Monitored temperatures during NATO experiment**. **Panel a:** waiting for handover to launch authority (between (1) and (2)), from handover to insertion into KUBIK, including launch and upload (between (2) and (3)), incubation in KUBIK (between (3) and (4)). **Panel b:** MELFI temperature during NATO cold stowage; spikes are due to door openings for handling of other hardware (HWs).


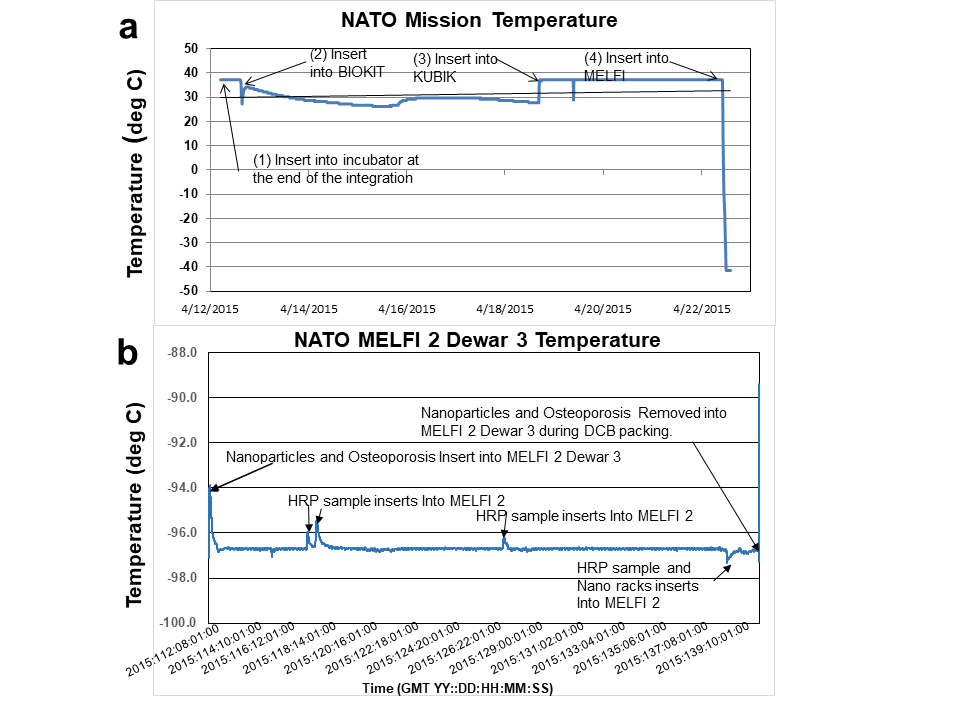


**Figure 5S**. **Qualitative analyses of RNA extracted from samples in the STROMA EU after their permanence onboard the ISS**. Evaluations were performed by the Agilent BioAnalyzer 2100.


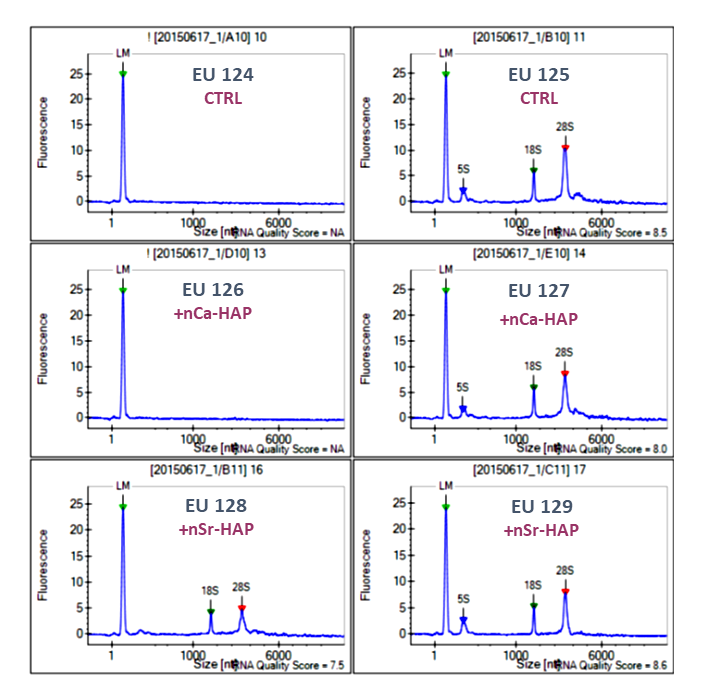


**Table 1S. Description of the main activities and objectives of the NATO project.**

| **NAnoparticles based countermeasures for Treatment of microgravity induced Osteoporosis (NATO).** | |
| --- | --- |
| NATO is a challenging collaborative project aiming to exploit the potential efficacy of Sr- and Ca-HAP on bone remodeling. | |
| **Involved Institutions** | **Main Scientific Activities** |
| University of Pavia  (PI) | - Synthesis and physicochemical characterization of nCa- and nSr-HAP. - *In vitro* biological characterization of the interactions of nCa- and nSr-HAP with osteoblasts and osteoclasts. - Set-up, optimization, and performing the flight experiment. - Preparation of the post-flight ground controls. |
| University of Milano  (Co-PI) | - *In vitro* biological characterization of the interactions of nCa- and nSr-HAP with osteoblasts and osteoclasts in RPM. - Testing the Experimental Units for the flight experiments. - Set-up, optimization, and performing the flight experiment. - Preparation of the post-flight ground controls. |
| Kayser Italia, Srl  (Co-Pi) | - Preparation of the Experimental Units for the flight experiments. - Experimental Sequence Test. - Performing the flight experiment. |
| Institute of Crystallography – CNR  (Co-PI) | - Mapping of the mineralization process by X-ray microdiffraction analyses on ground samples - Differential gene expression analyses by RT-PCR on samples treated or not with nCa- and nSr-HAP in 1g and simulated microgravity conditions. - Set-up, optimization, and performing the flight experiment. - Post-flight global gene expression analyses on flown and ground control samples by RNAseq. |
| The achievements will bring benefits in  **Science &Technology**  reducing the bone tissue resorption due to long stay on ISS  **Society**  reducing the healthcare costs and improving the astronauts’ quality of life  **Economy**  fostering technological transfer to industry | |

**Table 2S. Primers used for qRT-PCR study.** Housekeeping gene: 18S.

| **Genes** | **Upstream primer forward 5’- 3’** | **Downstream primer reverse 5’- 3’** |
| --- | --- | --- |
| *ALP* | ACCTCGTTGACACCTGGAAG | CCACCATCTCGGAGAGTGAC |
| *IBSP* | GGGCAGTAGTGACTCATCCG | TCAGCCTCAGAGTCTTCATCTTC |
| *COL1A1* | TGTAAGCGGTGGTGGTTATG | GGTAGCCATTTCCTTGGAAG |
| *COL3A1* | TGGATCAGATGGTCTTCCA | TCTCCATAATACGGGGCAA |
| *DCN* | CGAGTGGTCCAGTGTTCTGA | AAAGCCCCATTTTCAATTCC |
| *RUNX2* | ACAGTAGATGGACCTCGGGA | ATACTGGGATGAGGAATGCG |
| *OCN* | GGCAGCGAGGTAGTGAAGAG | CTGGAGAGGAGCAGAACTGG |
| *18S* | GTAACCCGTTGAACCCCATT | CCATCCAATCGGTAGTAGCG |

**Table 3S. Protein amount extracted from both ISS space flight and GC STROMA EUs expressed as µg/mL.** Data are presented as the average ± standard deviations for three measurements of the same sample**.**

|  | **# EU** | **Protein amount (µg/mL)** |
| --- | --- | --- |
| **ISS Samples** | 124 | 135.57 ± 0.41 |
|  | 125 | 103.67 ± 0.51 |
|  | 126 | 162.15 ± 0.91 |
|  | 127 | 116.96 ± 0.7 |
|  | 128 | 177.43 ± 1.02 |
|  | 129 | 182.08 ± 0.71 |
| **GC Samples** | 116 | 82.40 ± 0.60 |
|  | 117 | 101.01 ± 1.52 |
|  | 118 | 158.82 ± 0.64 |
|  | 119 | 164.14 ± 0.91 |
|  | 120 | 210.66 ± 0.41 |
|  | 121 | 174.11 ± 0.36 |
